# Late-Onset NR0B1-Related Adrenal Hypoplasia Congenita Presenting With Primary Adrenal Insufficiency and Pubertal Delay

**DOI:** 10.7759/cureus.98859

**Published:** 2025-12-09

**Authors:** João Filipe Nico, Rita Cardoso, Alice Mirante

**Affiliations:** 1 Pediatric Endocrinology, Diabetes and Growth Unit, Ambulatory Pediatric Department, Hospital Pediátrico de Coimbra, Unidade Local de Saúde de Coimbra, Coimbra, PRT

**Keywords:** adrenal hypoplasia congenita, delayed puberty (dp), hypogonadotropic hypogonadism, nr0b1, primary adrenal insufficiency

## Abstract

Primary adrenal insufficiency (PAI) in childhood is a rare condition most often caused by genetic defects rather than autoimmune disease. Among these, *NR0B1*-related adrenal hypoplasia congenita (AHC) is a distinctive X-linked disorder that may present beyond infancy and occasionally mimic congenital adrenal hyperplasia (CAH). We report the case of a 13-year-old boy previously diagnosed with CAH at the age of four because of chronic fatigue. On referral, he was receiving prednisolone and fludrocortisone and presented with short stature, absent puberty, and low adrenocorticotropic hormone (ACTH) levels. Switching to hydrocortisone improved biochemical parameters, but adrenal androgen precursors remained suppressed, and no pubertal development occurred by 15 years. AHC was suspected, and genetic testing revealed a hemizygous *NR0B1* pathogenic variant (c.1292del, p.(Ser431Ilefs*6)), previously reported only in early-onset disease. Testosterone therapy induced pubertal onset. Family history identified a maternal half-brother diagnosed in infancy with presumed CAH and a similar biochemical profile, and another asymptomatic infant half-brother, both referred for genetic evaluation. This case expands the clinical spectrum of *NR0B1*-related AHC, demonstrating that it can present as late as adolescence and be misdiagnosed as CAH. Persistently low adrenal androgens despite elevated ACTH serve as a diagnostic clue. Early genetic testing allows accurate diagnosis, individualized therapy, and appropriate counseling of at-risk families.

## Introduction

Primary adrenal insufficiency (PAI) in childhood is a rare but potentially life-threatening condition caused by deficient production of glucocorticoids, mineralocorticoids, or both. Unlike adults, in whom autoimmune adrenalitis predominates, most pediatric cases are genetic in origin [1]. Among these, *NR0B1*-related adrenal hypoplasia congenita (AHC) represents a distinctive X-linked disorder characterized by combined adrenal insufficiency and hypogonadotropic hypogonadism (HH) due to loss-of-function variants in the *NR0B1* gene encoding the DAX-1 protein [2,3].

The clinical spectrum of *NR0B1*-related AHC is remarkably heterogeneous. While most affected males present in infancy with salt-wasting crises, some develop adrenal failure later in childhood or adolescence, often with subtle symptoms such as fatigue, growth impairment, or delayed puberty [1,2,4,5]. This variability often delays diagnosis and can lead to misclassification as 21-hydroxylase deficiency, the most common cause of congenital adrenal hyperplasia (CAH) [1,6].

Biochemically, low adrenal androgens despite elevated adrenocorticotropic hormone (ACTH) contrast with the androgen excess typical of CAH [6]. Recognition of this discrepancy is crucial for prompting genetic investigation. Advances in molecular diagnostics have improved identification of the underlying defects, allowing accurate diagnosis, family counseling, and early initiation of appropriate hormone replacement [1,2,7].

We report the case of an adolescent boy with *NR0B1*-related AHC presenting with PAI and delayed puberty, initially misdiagnosed as CAH. This case illustrates the diagnostic pitfalls of late-onset *NR0B1* variants and underscores the importance of maintaining clinical suspicion for genetic causes of PAI beyond infancy.

## Case presentation

A 13-year-old migrant boy was referred to our pediatric endocrinology unit for follow-up of PAI. According to his mother, he had been diagnosed with CAH at age four because of persistent fatigue, although no investigation records were available.

At presentation, he was on prednisolone (3.9 mg/m²/day) and fludrocortisone (300 µg/day). In his medical history, short stature and occasional infections requiring temporary increases in glucocorticoid dosage were reported. There was no history of salt-wasting crises or hospitalizations. Family history revealed a four-year-old maternal half-brother who had been diagnosed with CAH at two months of age with a salt-wasting crisis and *Escherichia coli* pyelonephritis. He also presented with severe short stature (height z-score: -3.12) and was referred to our unit for follow-up.

On examination, the index patient had no hyperpigmentation, and the blood pressure was normal. Anthropometric evaluation revealed a weight of 28.4 kg, height of 127 cm (z-score: -4.34), and body mass index of 17.61 kg/m² (z-score: -0.55). No signs of pubertal development were noted.

Baseline laboratory evaluation showed low levels of ACTH (<5.0 pg/mL; reference: 9-52 pg/mL), cortisol (0.6 µg/dL; reference: 5-25 µg/dL), aldosterone (16.0 pg/mL; reference: 7-76 pg/mL), and dehydroepiandrosterone sulfate (DHEA-SO₄ <0.15 µg/mL; reference: 0.45-3.85 µg/mL), with normal insulin-like growth factor 1 (IGF-1 216 ng/mL; reference: 177-507 ng/mL) (Table 1). Bone age assessment revealed a marked delay, corresponding to seven years (Greulich and Pyle method).

**Table 1:** Biochemical evolution during follow-up. All hormonal measurements were performed in the same accredited clinical laboratory, using age-appropriate reference intervals. Time points marked with a superscript symbol (†, ‡, §) correspond to measurements obtained after a change in assay provider and reference intervals. Na = sodium; K = potassium; FSH = follicle-stimulating hormone; LH = luteinizing hormone; DHEA-SO₄ = dehydroepiandrosterone sulfate; ACTH = adrenocorticotropic hormone; IGF-1 = insulin-like growth factor 1; RR = reference range

Age	13 years 1 month	13 years 8 months	14 years 1 month	14 years 11 months	15 years 7 months	RR
Glucose (mg/dL)	77	83	73	87	68	60–100
Na (mmol/L)	141	141	139	140	139	136–146
K (mmol/L)	3.8	5.1	4.4	4.3	4.9	3.5–5.1
FSH (mUI/mL)	-	1.0	1.3	1.1	1.3	<15
LH (mUI/mL)	-	<0.1	0.2	0.2	0.4	<9.0
Total testosterone (ng/mL)	-	<0.1	<0.1	<0.1	1.4	Tanner stage I: 0.02–0.29; Tanner stage II: 0.04–2.79
DHEA-SO_4_ (µg/mL)	<0.15	<0.15	-	<0.15	<0.15	0.45–3.85
17-Hydroxyprogesterone (ng/mL)	-	-	0.04	0.04	0.04	0.29–2.06
ACTH (pg/mL)	<5.0	731	1099	420^†^	492^†^	9–52; ^†^7.2–63.3
Cortisol (µg/dL)	0.6	1.3	2.8	5.4	7.9^‡^	5–25; ^‡^3.7–19.4
IGF-1 (ng/mL)	216	220	157	337^§^	299^§^	177–507; ^§^125–503
Active renin (µUI/mL)	1.1	3.1	<0.5	17	48	7–76
Aldosterone (pg/mL)	16.0	23.0	16.5	29.1	36.7	40–310

Prednisolone was switched to hydrocortisone 10 mg/m²/day in three divided doses, and fludrocortisone was reduced to 50 µg/day (Table 2). Screening for *CYP21A2* gene mutations by multiplex ligation-dependent probe amplification was negative in both patients.

**Table 2:** Treatment plan during follow-up.

Age	13 years 1 month	13 years 8 months	14 years 1 month	14 years 11 months	15 years 7 months
Prednisolone (mg/m^2^/day)	3.9	-	-	-	-
Hydrocortisone (mg/m^2^/day)	-	10	9	13	12.5
Fludrocortisone (µg/day)	300	50	50	50	50
Testosterone (mg/month)	-	-	-	-	25

During follow-up, the hydrocortisone dose was increased due to persistently elevated ACTH and low cortisol levels. Despite elevated ACTH, 17-hydroxyprogesterone, DHEA-SO₄, and androstenedione levels remained low (Table 1).

At 14 years and 11 months, he remained prepubertal, with low testosterone (<0.1 ng/mL), follicle-stimulating hormone (1.3 mIU/mL), and luteinizing hormone (0.2 mIU/mL), consistent with HH (Table 1). His height z-score improved to -3.71, and bone age was 8.5 years.

Given the combination of PAI and HH, AHC was suspected. Next-generation sequencing using the AmpliSeq™ methodology (Illumina) identified a hemizygous pathogenic variant in exon 2 of the *NR0B1* gene (c.1292del, p.(Ser431Ilefs*6)), confirming the diagnosis.

At 15 years of age, testosterone therapy was initiated at 25 mg intramuscularly once monthly. After six months of treatment, pubertal progression to Tanner stage II was observed (testicular volume: 5-6 mL bilaterally and sparse pubic hair). At this point, the height z-score was -3.53, and the testosterone dose was increased to 50 mg monthly.

**Figure 1:**
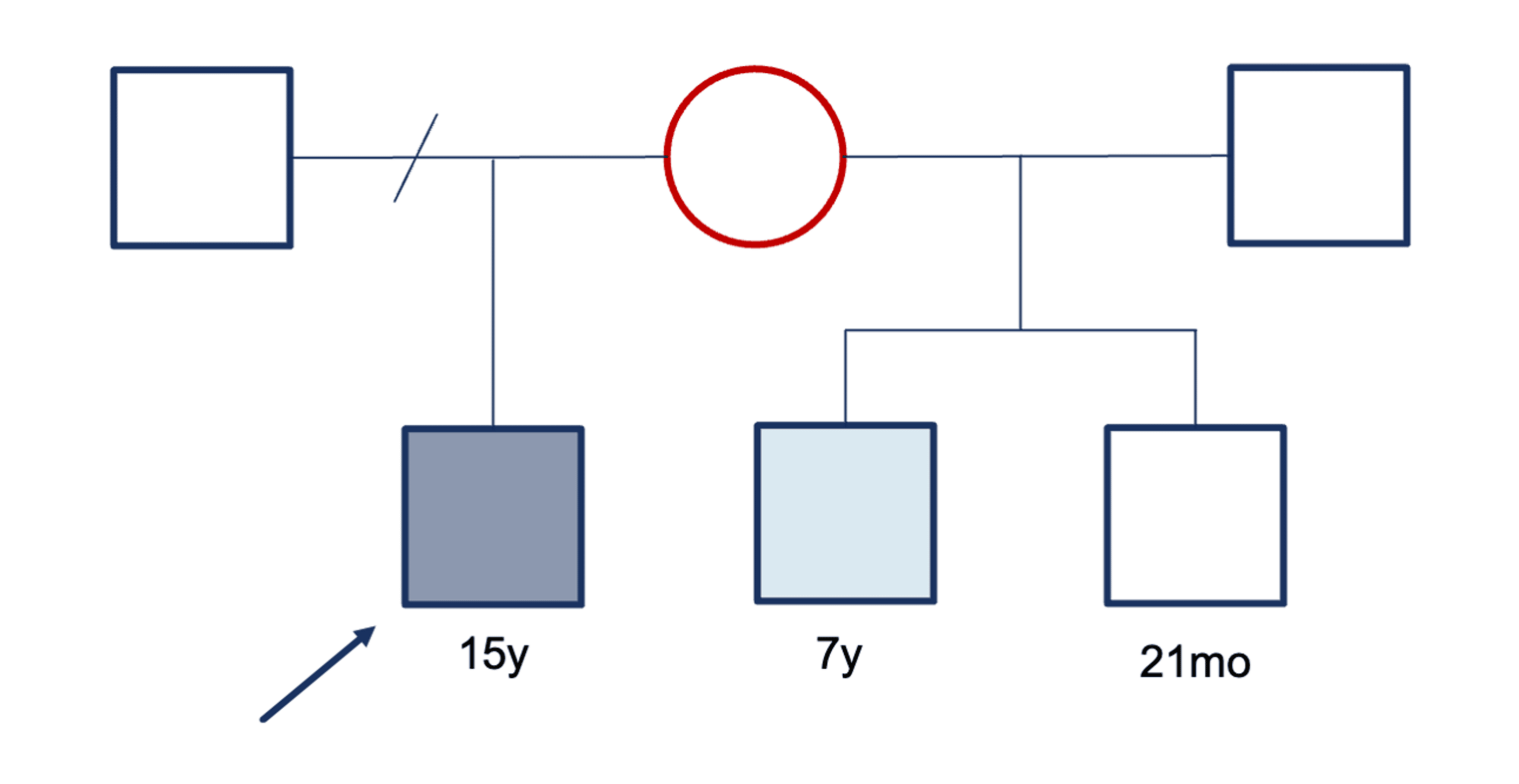
Family pedigree of the proband with NR0B1-related adrenal hypoplasia congenita. The proband (arrow, 15 years) carries a hemizygous *NR0B1* pathogenic variant (c.1292del, p.(Ser431Ilefs*6)). His mother (red circle) is an obligate heterozygous carrier. The maternal half-brother (light blue square, seven years) shows a similar biochemical profile but is pending genetic confirmation. The youngest maternal half-brother (white square, 21 months) remains asymptomatic with normal biochemical results and is also awaiting genetic testing. Red circle = carrier female; dark blue square = affected male; light blue square = suspected affected male; white square = unaffected male.

During follow-up, the mother gave birth to another maternal half-brother, who was asymptomatic at 21 months of age with normal biochemical results. The older maternal half-brother developed biochemical findings closely resembling those of the index case (Figure 1). Genetic testing for AHC was pending for both half-brothers at the time of this report, and the family was referred for genetic counseling.

## Discussion

This report describes an adolescent male with PAI and pubertal delay due to a pathogenic *NR0B1* variant, broadening the phenotypic spectrum of AHC. The persistently suppressed adrenal androgens despite elevated ACTH and the coexistence of HH were key diagnostic clues distinguishing this condition from 21-hydroxylase deficiency. Genetic analysis confirmed a hemizygous *NR0B1* variant (c.1292del, p.(Ser431Ilefs*6)), previously reported only in early-onset adrenal failure [8,9], but not in late presentations.

*NR0B1*-related AHC is a rare X-linked disorder accounting for a minority of pediatric PAI cases [2,3]. Most affected boys present in early infancy with salt-wasting crises due to complete adrenal failure [1,2,8]. However, partial or delayed forms may appear later in childhood or adolescence, often with insidious fatigue, growth impairment, and delayed puberty [1,2,9]. Such milder presentations can be misclassified as CAH or isolated HH, as occurred in our patient. Persistent suppression of adrenal androgen precursors despite high ACTH should prompt suspicion of AHC rather than 21-hydroxylase deficiency [6].

The initially low ACTH likely reflected supraphysiologic exposure to prednisolone, a long-acting glucocorticoid that suppresses pituitary secretion and can impair growth [4,6]. Switching to hydrocortisone and adjusting fludrocortisone improved biochemical control and growth, consistent with current pediatric recommendations [4,6]. Despite improved control, intermittent ACTH elevation suggested variable adherence or incomplete replacement, a common challenge in adolescents with chronic endocrine disorders.

The *NR0B1* gene, located on chromosome Xp21, encodes DAX-1, a nuclear receptor essential for adrenal development and regulation of the hypothalamic-pituitary-gonadal axis [2,3]. The frameshift variant identified introduces a premature stop codon, leading to loss of repressor function [8,9]. Although previously linked to early disease, its late presentation suggests residual DAX-1 activity or modifying genetic or environmental influences [2]. The heterogeneity of *NR0B1* variants is well documented, ranging from neonatal adrenal crisis to adult-onset HH [2,9,10].

Family history provided a crucial diagnostic context. One maternal half-brother, diagnosed in infancy with presumed CAH, displayed a similar biochemical profile but tested negative for *CYP21A2* mutations. The youngest half-brother remains asymptomatic with normal results but under follow-up, as a delayed onset cannot be excluded. Given the X-linked inheritance, the mother is likely a heterozygous carrier. These findings underscore the importance of cascade genetic testing to identify affected relatives and enable early intervention [1,2,7].

Beyond diagnosis, *NR0B1*-related AHC carries long-term reproductive implications. Even with adequate androgen replacement, spermatogenesis is often impaired, and spontaneous fertility is rare. Nevertheless, successful paternity through assisted reproductive techniques has been reported [10], emphasizing the need for early counseling and lifelong follow-up.

The strengths of this report include detailed longitudinal observation, integration of biochemical and genetic data, and documentation of a late-onset phenotype for a variant previously linked only to early disease. Limitations include the absence of functional assays to confirm residual DAX-1 activity and incomplete segregation analysis, as family testing was ongoing.

## Conclusions

This case broadens the clinical spectrum of *NR0B1*-related AHC, demonstrating that the c.1292del (p.Ser431Ilefs*6) variant can manifest as late as adolescence. Persistently low adrenal androgens despite elevated ACTH are a key diagnostic clue distinguishing this condition from CAH. Early recognition and genetic testing enable accurate diagnosis, individualized hormone replacement, and family counseling. Identification of vertical transmission through cascade testing highlights the importance of genetic screening in apparently isolated cases and supports long-term follow-up of at-risk males.
